# Application of CRISPR-Cas9 Based Genome-Wide Screening Approaches to Study Cellular Signalling Mechanisms

**DOI:** 10.3390/ijms19040933

**Published:** 2018-03-21

**Authors:** Sumana Sharma, Evangelia Petsalaki

**Affiliations:** 1 European Bioinformatics Institute, European Molecular Biology Laboratory, Wellcome Genome Campus, Hinxton, Cambridge CB10 1SD, UK; petsalaki@ebi.ac.uk; 2 Cell Surface Signalling Laboratory, Wellcome Sanger Institute, Wellcome Genome Campus, Hinxton, Cambridge CB10 1SD, UK

**Keywords:** CRISPR/Cas9, genome-wide screens, cellular signalling

## Abstract

The cellular signalling process is a highly complex mechanism, involving multiple players, which together orchestrate the cell’s response to environmental changes and perturbations. Given the multitude of genes that participate in the process of cellular signalling, its study in a genome-wide manner has proven challenging. Recent advances in gene editing technologies, including clustered regularly-interspaced short palindromic repeats/Cas9 (CRISPR/Cas9) approaches, have opened new opportunities to investigate global regulatory signalling programs of cells in an unbiased manner. In this review, we focus on how the application of pooled genetic screening approaches using the CRISPR/Cas9 system has contributed to a systematic understanding of cellular signalling processes in normal and disease contexts.

## 1. Introduction

**Figure 1:**
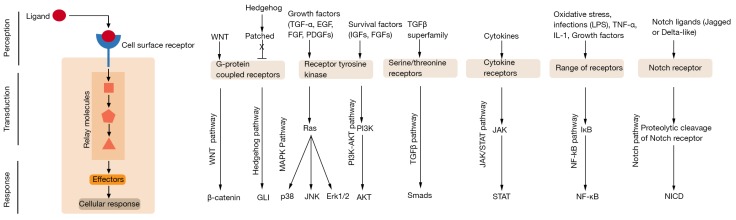
Overview of major cellular signalling process and pathways. Cell surface receptors receive instructional signals from the environment through interaction with extracellular ligands. This signal is then transduced across the plasma membrane where intracellular second messengers amplify and relay the information to elicit cellular responses. Ligands that utilise common signalling pathways and associated transcription factors are depicted in the left panel (an excellent resource for an overview of cellular signalling pathways can be found at the Internet resource: Cell Signaling Pathways by Michael Berridge [12]).

Cellular signalling is pivotal for a number of fundamental biological processes, including growth, proliferation, differentiation and programmed cell death [1]. Cellular signalling processes are typically initiated by cell surface receptors embedded on the plasma membrane when they receive extracellular signalling cues either via soluble factors (e.g., secreted proteins, hormones, autacoids and neurotransmitters) or through direct cell-cell interactions between specific receptors exposed on the outer surface of neighbouring cells. The intracellular domain of membrane receptors is in direct contact with the components of the cellular cytoplasm, which allows membrane proteins, such as G-protein linked receptors (GPCRs), enzyme-linked receptors and ion channels, to function as `signal transducers’ to transmit extracellular signals across the membrane. Membrane proteins that transduce the extracellular signal use different signalling pathways (Figure 1) in a cell to generate complex regulatory networks. Defects in the cell signalling pathway are associated with a multitude of human diseases [2]; therefore, there is considerable interest in understanding the cell signalling pathways in the normal and disease context for effective design of therapeutic targets.

It is now widely appreciated that the cell signalling process is not a discrete linear flow of information, but rather an interconnected network involving many players. A cell receives a variety of instructional cues from the environment, which it then integrates via communication between distinct signalling pathways in order to relay information for a cogent biological response [3]. The sheer number of players in the cellular signalling pathway, the functional redundancies between them and the crosstalk between these players have made the systematic dissection of cellular signalling pathways challenging. Pioneering studies in the model organism *S. cerevisiae* (yeast) have used a diverse set of methods in order to generate a global profile of the cell state, using proteomics, phosphoproteomics, RNA-Seq-based approaches, chromatin immunoprecipitation followed by sequencing (CHIP-Seq) and combining these with computational approaches and perturbation studies in order to generate a signalling network for a given physiological context [4,5,6]. A comprehensive study of cellular signalling and its biological outcome in cells requires utilization of both experimental and computational approaches. Common approaches to characterize the global signalling network of a cell involve methods such as phosphoproteomics profiling (reviewed in [7]) and reverse-phase protein arrays [8]; however, such methods are generally either low throughput or measure only a small subset of the phosphoproteome. In addition, the technical difficulties associated with these methods often create inconsistent results. On the other hand, systematic perturbations of genes provide direct causal evidence and have always constituted powerful tools with which to study cellular networks, but the technological shortcomings in manipulating the diploid human genome have limited the use of such techniques. RNA interference (RNAi) technology provided a platform for high throughput gene silencing of mammalian genomes through sequence-specific targeting of mRNA; however, one of the biggest challenges of using RNAi as a tool to study gene function involved the sequence-specific off-target effects of siRNA [9]. The genetic perturbation using RNAi frequently resulted in incomplete silencing, which, combined with the off-target effects, often led to a decrease in sensitivity and inconsistent results [10]. Recent technological advancements in genome-editing technologies using the clustered regularly-interspaced short palindromic repeats/Cas9 (CRISPR/Cas9) (see the review on CRISPR technology [11]) now serve as an alternative powerful means for conducting forward genetic screens to study a biological system in a genome-wide manner, which is ideal for the unbiased investigation of intricate cellular signalling networks. In this review, we focus on the CRISPR/Cas9 approach to conduct large-scale pooled perturbation-based studies to study cellular signalling pathways. We will mainly concentrate on the studies that have interrogated different aspects of the cellular signalling processes with pooled screens conducted using viability-based and marker-based selection approaches.

## 2. Different Strands of Pooled CRISPR Screens

There are several approaches to performing pooled genetic screens using the CRISPR/Cas9 technology (see the reviews [13,14]). A pooled screening approach provides an opportunity to interrogate thousands of genetic perturbations in a single experiment. A pooled screen using the CRISPR/Cas9 system starts with the generation of a library of perturbed cells using a library of gRNAs. The gRNA, which is usually delivered via a lentivirus or other retrovirus integrating into the genome of the cells, serves as a molecular tag. The cells can then be separated according to the phenotype of interest, and the genes causing the phenotype can be read out by first isolating genomic DNA from the cell population using PCR followed by massive parallel sequencing (using next-generation sequencing (NGS)) across the gRNA-encoding regions, then mapping each sequencing read to a pre-compiled list of the designed gRNA library. Computational analysis such as MAGeCK (Model-based Analysis of Genome-wide CRISPR-Cas9 Knockout) [15], BAGEL (Bayesian Analysis of Gene EssentiaLity) [16] and caRpools (CRISPR AnalyzeR for Pooled Screens) [17] can then be used in order to determine the differences in the abundance of gRNAs between the control and the phenotyped sample, thereby allowing for the identification of genes responsible for the observed phenotype. Given the ease of generating libraries containing thousands of gRNAs that can be used to create large mutant collections, the CRISPR/Cas9 technology has quickly become the method of choice for large-scale pooled screens. The majority of the pooled screens performed thus far have used the wild-type (WT) Cas9 to perform CRISPR-KO screens. However, an increasing number of studies now also utilize the catalytically-dead mutant of Cas9, referred to as dead-Cas9 or dCas9, in which the nuclease activity of the WT Cas9 has been mutated in order to render it non-functional. dCas9 has been fused to a range of chromatin modifier fusion proteins to convert it into a highly versatile enzyme that can be used to perform activation (CRISPR activation (CRISPRa)) or repressional (CRISPR interference (CRISPRi)) screens [18]. We will now review in turn the basic concepts underlying different styles of pooled screening approaches, followed by studies that have been performed to date, which employ such screening strategies to investigate the cellular signalling process.

### 2.1. Viability-Based Negative-Selection Screens

The purpose of a negative selection screen is to identify perturbations that affect the survival or proliferation of cells, which cause the perturbed cells to be depleted during selection. The approach here is to transduce two sets of cell populations and subject one set to the selection while the other serves as a non-selected control. The gRNA abundance in both populations is then analysed in order to identify gRNAs that have been depleted because of the selection. One of the simplest forms of negative selection screen is the continued growth of cells for an extended amount of time to identify genes that are required for the proliferation of cells. Such screens have been used to identify both essential genes that are required for cell lines tested and a small set of genetic dependencies of specific cancer cell lines [19,20,21,22,23].

CRISPR-based gene essentiality screens can be used to identify signalling pathway dependencies of different cancer cell lines depending on their mutational background. Cancer cells often have alterations in the different aspects of the signalling pathways, which lead to escape from normal cellular mechanisms that keep proliferation under control. Dysregulation in processes such as growth, migration, differentiation and motility is often a result of mutations in proto-oncogenes or oncogenes, which cause hyper-activation of the signalling pathways they control. Cancer cells often become dependent on the activity of such oncogenes [24]. Examples of oncogenic mutations in the context of cellular signalling include mutations in growth factor receptor tyrosine kinases (RTKs, e.g., EGFR), monomeric GTP-binding proteins (e.g., Ras family proteins KRAS, NRAS and HRAS), serine/threonine kinases, cytoplasmic tyrosine kinases (e.g., Raf, AKT), lipid kinases (phosphoinositide 3-kinases, PI3Ks), nuclear receptors (thyroid and steroid hormone receptors) and nuclear transcription factors (Myc, Fos/Jun and Rel) [25].

Essential gene screens performed by Hart et al. allowed for the correct identification of the dependency of the Mitogen-activated protein kinase (MAPK) pathway in cell lines that harbour the *KRAS* or *BRAF* driver mutations and dependence upon E3 ubiquitin-protein ligase (MDM2) on cells that carry the WT *TP53* [20]. Similarly, a study by Tzelepis et al. was also able to identify correctly the dependence of NRAS on acute myeloid leukaemia (AML) cell lines carrying activating mutations in this gene [19]. A negative selection genome-wide forward genetic screen using the CRISPR-Cas9 KO approach was carried out in E3 ubiquitin-protein ligase RNF43-mutant pancreatic ductal adenocarcinoma (PDAC) cells, which are dependent on the wnt signalling pathway to correctly identify the WNT signalling pathway as an essential component for cellular proliferation [26]. In this study, the authors further identified a single member of the frizzled family gene, *FZD5*, as an essential gene only in the context of RNF43 mutational background. The authors pointed out that although the human genome contains 19 WNT-family ligands and 10 WNT receptors (or the frizzled gene family), the finding that only one out of ten Wnt receptors (FZD5) was enough to drive the proliferation of the cells in this particular context underlines the importance of context-dependent receptor-ligand interaction specificity of signalling processes that can be harnessed to devise targeted therapies.

Loss-of-function negative selection screens performed in cell lines with a given genetic background can constitute the basis of the identification of synthetic lethal interactions, whereby simultaneous perturbation of two genes results in loss of cellular viability (reviewed in [27]). The identification of synthetic interactions has the potential to open up new means of cancer treatment, as it allows for the targeted treatment of cancer cells, making only cells with a particular mutation susceptible to the drug in question. Wang et al. have recently carried out a comprehensive study using 14 AML cell lines to identify synthetic lethal interactions with oncogenic Ras [28]. By using a panel of three mutant NRAS and three mutant KRAS cell lines and comparing their essential gene profiles with those of six AML cell lines with WT Ras, the authors were able to identify five genes, the inactivation of which conferred lethality only in Ras-dependent lines. These included genes responsible for post-translational modification of Ras proteins—*RCE1* and *ICMT*, genes in the MAPK signalling pathway—*RAF1* and S*HOC2* and a guanine nucleotide exchange factor (GEF) for the Rac GTPases named *PREX1*. Unlike the other identified hits, *PREX1* was found to be essential only for Ras-driven AML, but not in the other cancer types that were tested, showing that synthetic interactions with oncogenes can be cell context-dependent. The authors further validated the hits in a comparative study using a WT murine pro-B cell line and an isogenic version of the same line with an oncogenic NRAS that exhibited a dependence on the MAPK signalling pathway.

The CRISPR-KO approach has been reported to perform better with low noise, minimal off-target effects and experimental consistency, especially in lethality-based essential gene screens when compared to knock down approaches using both CRISPRi and short-hairpin RNA [29]. That said, the application of CRISPR-based KO screens to identify essential genes does have its limitations. The use of WT Cas9 does not always lead to KO as there is always a possibility that the indels that are created by the cellular repair machinery to repair the double strand breaks (DSBs) might generate an in-frame mutation, thereby keeping the gene function intact. This can lead to the generation of a mixed population, thereby making the screen `noisy’ and the interpretation of data challenging. In addition, a number of studies has shown that the higher the number DSBs in a cell, the higher the chance of cellular lethality independent of the gene being targeted. Therefore, CRISPR KO-based screens can falsely identify genes within highly amplified regions, including non-expressed genes as essential genes [30,31]. Recently, Mayers et al. have developed a computational method, CERES, that takes into account the copy-number effect to estimate gene-dependency levels in KO-based essentiality screens [32]. In this study, CRISPR/Cas9 KO screens were performed in 342 cancer cell lines representing 27 cell lineages, and the hits obtained from the screen were corrected using the computational method. The authors showed that the correction model did not hinder the identification of known cancer-specific dependencies in amplified regions as they were able to identify the KRAS dependence of a KRAS-mutant pancreatic cancer cell line in which the gene was amplified. Such large-scale KO screening projects where a large number of cancer cells is screened aim to identify all genetic dependencies of all cancer cells and will constitute an important contribution to the field of cellular signalling processes.

### 2.2. Viability-Based Positive-Selection Screens

In a positive selection screen, a strong selective pressure is introduced, such that the probability of cells being selected without the genetic perturbation is low. These screens have been used to identify perturbations that confer resistance to drugs [33,34], toxins [35] and pathogen infections [36,37,38,39,40,41,42] (also reviewed in [43]). Unlike negative selection screens, the signal for a positive selection is usually strong, as the abundance of relevant gRNAs in such screens increases relative to the rest of the gRNAs, which allows for the easy detection of resistant cells.

Multiple studies have carried out positive selection screens in cell lines that have altered signalling pathways. In one of the pioneering CRISPR-KO screens by Shalem et al., a positive selection screen was carried out in a melanoma cell line, A375, which harbours a highly frequent BRAFV600E mutation, to identify known and novel factors that confer resistance to vemurafenib, a drug that inhibits the mutant kinase BRAF [33]. Similarly, Krall et al. have also carried out a similar study to identify factors that confer resistance to RTK/Ras/MAPK inhibition in lung cancer [44]. The authors were able to identify multiple factors that conferred resistance to the different drugs; however, they focused their study on KEAP1, as it was found to modulate sensitivity to EGFR, ALK, BRAF or MEK (MAPKK/ERK) inhibition in lung cancer cell lines with a range of mutational backgrounds, which included *EGFR, ALK, BRAF, KRAS* or *NRAS* mutations. *KEAP1* was also identified in a separate genome-wide KO screen carried out by Terai et al. for the identification of factors conferring resistance to tyrosine kinase inhibitors on a EGFR-dependent cell line [45]. The main focus of this study, however, was to identify factors that modulate resistance to combination therapies that utilize both tyrosine kinase inhibitors and transcriptional inhibitors in lung adenocarcinoma and other cancer treatments. The authors identified the components of the cellular ufmylation pathway (ligation of a Ubiquitin-fold modifier 1 (UFM1) to a substrate) whose loss can lead to protective unfolded protein response, leading to pro-tumourigenic inflammatory response that confers drug resistance through ER stress rather than through the MAPK signalling pathway.

A complementary approach to identify genes required for the resistance of A375 melanoma cell lines to vemurafenib was performed by Konnerman et al. in a genome-wide CRISPR activation rather than KO screen [46]. The paper first described the CRISPR-activation screen-based dCas9-synergistic activation mediator (SAM) system that uses dCas9 fused with VP64 and an aptamer-modified sgRNA that contains two binding sites for the modified version of a bacteriophage coat protein MS2. MS2 itself is used as a fusion protein with two other activator helper proteins, NF-κB trans-activating subunit p65 and human heat-shock factor 1 (HSF1). The SAM activation system was then used to conduct a genome-wide activation screen in drug-sensitive A375 melanoma cell lines to identify factors whose expression causes resistance to drug treatment. The screen was able to identify the components of the ERK signalling pathway, multiple GPCRs that signal through the ERK, PI3K, cAMP and PKA pathways and ITG receptor family proteins that interact with receptor tyrosine kinases (RTKs) to activate ERK and PI3K signalling cascades.

Wang et al. have recently utilized a positive selection screen on two KRAS-mutant pancreatic cancer cell lines to identify factors that confer survival and proliferation to trametinib, a drug that inactivates the MAPK pathway through inactivation of MEK [47]. Trametinib is a commonly-used drug for KRAS- or BRAF-mutant cancers, and by using a moderate dose of the drug, for which only 50% of the cells showed a decreased cell proliferation, the authors were able to identify CIC as a factor that promoted proliferation and survival of KRAS-mutant cells upon drug treatment. The authors validated the role of CIC by follow-up studies, in which CIC was identified as the transcriptional repressor of the PEA3 family of ETS transcription factors (ETV1, ETV4 and ETV5), and the over-expression of these transcriptional factors in the context of KRAS-mutation was found to be sufficient to reduce sensitivity to MEK inhibition. Furthermore, deletion of ATXN1L, a factor that forms a complex with CIC and enhances the CIC transcriptional repressor activity, was also found to reduce sensitivity to MEK inhibition. Positive selection screens are often restricted to the identification of non-essential genes or non-redundant genes, which was pointed out by the authors in this study, in which they were unable to identify other negative regulators of the MAPK pathway that potentially have functionally redundant family members.

A similar approach was also very recently utilized by Kong et al. in the BRAFV600E melanoma cell line, but rather than investigating the genes responsible for conferring resistance to treatment with drugs, the authors studied the process of drug addiction in cancer cells [48]. In this approach, cell lines that were resistant to the BRAF inhibitor (BRAFi) dabrafenib or dabrafenib in combination with the MEK inhibitor (MEKi) trametinib were first generated by longitudinal treatment of sensitive cell lines in the presence drugs over a period of 3–5 months. The resistant clones generated were addicted to the drugs, as discontinuation of the drugs caused these cells to die. A genome-wide KO screen was next conducted on the drug-addicted cell lines, which allowed for the identification of well-known components of the signalling pathway, ERK2 kinase and transcription factor JUNB, which were responsible for conferring the drug addiction phenotype. Further in vitro and in vivo experiments were used to identify ERK2, JUNB and a JUNB binding partner protein, FRA1, as the critical components required for this phenotype. Interestingly, the pathway for this drug addiction was the same for all cells that were tested, regardless of how drug resistance had developed in a particular cell in the first place. This study also opened up interesting questions regarding the complexity of the signalling process, as signalling through ERK2, but not ERK1 was shown to be crucial for the drug addiction phenotype, even though both are critical components of the MAPK pathway.

A requirement for a viability-based pooled approach is that the condition of study should lead to proliferation or death of cells, such that mutants that are either enriched or depleted upon addition of the condition can be effectively selected. However, there are many cellular signalling processes that do not lead to a change in cellular viability, and for such processes that do not have a selectable phenotype, viability-based pooled screening methods can be restrictive. That said, a viability-based CRISPR knock-out screening to study the Hedgehog signalling pathway for ciliary signalling that intrinsically lacks a viability phenotype has been performed by Breslow et al. In this study, the authors generated a reporter line that expresses the blasticidin resistance cassette via the GLI transcription factor response element upon Hedgehog signalling [49]. This essentially allowed them to perform a viability-based screen to identify positive and negative regulators of the pathway by identifying the gRNAs that were either depleted or enriched after blasticidin treatment of a blasticidin-sensitive reporter line. The authors identified almost all known regulators of the pathway and novel components in the ciliary structure, including a protein complex required for centriole maintenance.

Recently, a novel screening system named as the `two cell type’ (2CT)-CRISPR assay has been developed and used by Patel et al. to identify genes on melanoma cell lines that are essential for susceptibility to T-cell-mediated killing [50]. In this system, genome-wide mutant populations of melanoma cell lines were subjected to effector T-cells, and cells that survived the killing were harvested in order to identify essential genes for cancer immunotherapy. The authors were able to identify over 100 genes that were involved in a number of cellular processes, ranging from the known antigen presentation and IFNγ signalling to previously unlinked pathways that are also important for cytotoxic T-cell-based immunotherapy, including EIF2 signalling, endoplasmic reticulum stress, apoptosis, assembly of RNA polymerase II, TNF receptor signalling and protein ubiquitination.

### 2.3. Marker-Based Selection Screen

A third type of selection is marker gene selection, in which the phenotype is not based on the lethality of the cells, but rather on mutations that change marker gene protein expression. In this type of screen, the marker gene is either endogenously-tagged with fluorescent proteins or labelled with highly specific antibodies; then, cells with gRNAs that target genes whose perturbations contribute to the expression of the marker gene are isolated using fluorescence-activity cell sorting (FACS)-based approaches.

Multiple studies have also made use of marker-based selection to identify regulators of specific genes or pathways of the cellular signalling system. Parnas et al. have used a KO screen to uncover the regulatory network in an innate immune system using primary mouse bone marrow-derived dendritic cells (DCs) [51]. In the study, the expression of tumour necrosis factor (Tnf) in DCs was used as a marker upon lipopolysaccharide (LPS) stimulation. A FACS-based sorting strategy was designed in the study, in which cells within the mutant population that were either refractory to Tnf expression or that induced Tnf more strongly were collected differentially and assessed for the cellular factors that mediated the regulatory response. A large number of genes was identified in the screen as important for the regulation of Tnf expression upon LPS stimulation; the highest ranking 176 (112 positive and 64 negative) candidate regulators were chosen for targeted gene KO validation. Of the tested genes, 57/112 positive, but only 4/64 negative regulators were correctly validated. To reduce the high number of false-positive genes, the authors opted for a secondary validation with a focused library targeting approximately 2500 genes identified in the primary screen, which was shown to improve the specificity and sensitivity of a pooled screen. The authors then categorized the hits into functional modules and uncovered components of the the oligosaccharyltransferase (OST) protein glycosylation complex and endoplasmic reticulum (ER) folding and translocation pathway and the PAF complex, which is involved in the regulation of transcriptional elongation as novel regulators of Tnf expression.

The marker-based screening approach using FACS has also been applied by DeJesus et al., who have used a CRISPR/Cas9-based genome-wide KO screen to study the regulators of SQSTM1, a protein that acts as a regulator of the nuclear factor-κB (NF-κB) signalling cascade and cellular autophagy [52]. In the study, a neuroglioma H4 cell line that stably expressed green fluorescent protein (GFP)-tagged SQSTM1 was used to perform a genome-wide KO screen with the CRISPR/Cas9 system to identify regulators of SQSTM1 based on the GFP fluorescence. A secondary focused screen was next carried out using staining of endogenous SQSTM1 to validate the findings from the primary genome-wide screen. The authors were able to identify the known macroautophagy machinery and the novel role of the UFMylation process as regulators of SQSTM1 protein expression. Another recent study by Potting et al. also utilized a similar approach to study the regulators of PARKIN abundance in a cell [53]. Here too, the authors generated a cell model that expressed a transgene encoding GFP-PARKIN under the control of a large PARK2 promoter fragment and performed genome-wide KO screens using a FACS-based sorting strategy to identify regulators of the protein at a steady state.

Pushupati et al. have also used a CRISPR/Cas9-based KO screen on the genome scale to identify regulators of the Sonic Hedgehog signalling pathway. In their approach, rather than fusing an effector protein with GFP, the authors generated a mouse fibroblast cell line, which expressed GFP from a Hedgehog-responsive promoter element [54]. The genes that regulate the signalling pathway were identified based on the GFP fluorescence upon treatment of a cell with different concentrations of the Sonic Hedgehog ligand (SHH). This study was able to identify both positive (genes regulating the ciliary function) and negative regulators (genes that suppress Smoothened (SMO) accumulation in primary cilia) of Hedgehog signalling. A similar strategy has also been used by Lebensohn et al.; they focused on the regulatory mechanisms in canonical WNT signalling pathway [55]. These authors also utilized a market-based FACS-based screening strategy using WNT reporter HAP1 haploid cell lines, which consist of WNT-responsive elements that drive the expression of EGFP upon WNT signalling activation. By using the expression of GFP as a proxy for the activation or inactivation of the signalling pathway, the authors were able to identify both known and novel regulators of this cell signalling pathway. The authors in this study used a gene-trap rather than a CRISPR/Cas9-based genome-wide screening approach to conduct their genome-wide screens.

These studies have demonstrated the way in which FACS-based CRISPR-KO screens can be used to carry out a comprehensive dissection of genetic pathways contributing to a specific protein expression in a defined cellular context. FACS-based screens are generally thought to have an advantage over lethality screens for the identification of genes that have intermediate phenotypes, as the quantitative nature of flow-cytometry allows for the selection of cells with mutations that result in a partial, as well as a complete phenotype. However, the studies that have used the FACS-based selection approach also highlight the importance of secondary validation steps that are usually required for high-confidence identification of genes.

### 2.4. Combinatorial Screens

Combinatorial screens allow detailed elaboration of how genes function together in complex genetic signalling networks. Loss-of-function genetic interaction screens can constitute powerful means to deconvolute the complex cellular signalling pathway, as already demonstrated by multiple RNAi-based screens that have performed pairwise perturbations of signalling factors in a single cell to identify positive and negative regulators of signalling pathways to generate a context-dependent genetic interaction network [56,57].

The advancements in the field of CRISPRi- and CRISPRa-based approaches together with the KO screens now provide a wider platform to conduct complex screens where genes can be knocked out, knocked down or even activated at the same time in a single cell [58,59,60,61,62,63]. Recently, an orthogonal CRISPR screening system that allows simultaneous activation and deletion of two genes in the same cell has been applied to identify genetic interactions between cancer-relevant genes. The authors first applied a CRISPRa-based gain-of-function screen to investigate cancer pathway genes using the K562 chronic myeloid leukaemia cell model [64]. In this approach, the mutant cell line over-expressing genes was subjected to a kinase inhibitor, imatinib, that specifically kills cells with a BCR-ABL oncogenic fusion. Cells within the drug-resistant population were isolated to identify genes that when over-expressed caused loss of drug sensitivity. The authors were able to identify 332 candidate genes that included previously-characterized imatinib resistance genes and genes whose over-expression in patients manifests high tolerance to imatinib. In addition, they were also able to identify components of the BCR-ABL signalling pathway including BCR-ABL binding partners and downstream effectors proteins. To further explore the interaction between the genes identified in this screen, a K562 cell line that expressed the *S. pyogenes*-based SunTagCRISPRa system [65] and Cas9 nuclease from *S. aureus* (SaCas9) was generated, and an orthogonal activation/knock-out screen to study the genetic interactions between the 87 genes identified in the primary screen was performed. Based on the validated interactions, the authors were able to create a directional Ras-centric genetic interaction model. Furthermore, the observed genetic interaction between the negative regulator of Ras, NF1 and the TAM receptor tyrosine kinase AXL was identified to be of therapeutic interest as cells lacking NF1 were found to be highly sensitive to the AXL inhibitor R428. The authors suggested that NF1-deficient cells become dependent on AXL signalling, which then makes them selectively targetable by the AXL inhibitors. This study showed how combinatorial screens can allow for the identification of rerouted signalling dependencies in cancer cells.

## 3. Conclusions and Further Directions

In recent years, the CRISPR/Cas9-based pooled screening approach has rapidly revolutionized the field of functional genomics. The ease of generation of mutants, the lower off-target effects and the engineered versatility of the Cas9 protein generally make genome-wide CRISPR/Cas9-based screens preferable to large-scale RNAi screens (see [66] for a review). That said, no matter the screening platform, any large-scale screening system will inherently identify false positives; therefore, it is necessary to be aware that pooled screens are only the beginning of a study and that downstream validation of `hits’ identified in such screens is crucial if screening systems are to be used to study biological processes. Computational tools and secondary validation screens using smaller focused libraries can be particularly useful to narrow the list of `hits’ that should be followed up in the subsequent studies.

The majority of the pooled CRISPR/Cas9 screens discussed in this review have focused on disease biology, specifically cancer biology. However, by carefully designing the screening parameters, for example by generating marker lines specific to the biological process of interest, other facets of cellular signalling processes such as differentiation, motility, proliferation, metabolism and immunity can also be studied with this tool (refer to Table 1 of [14]). We have recently been working on devising a genome-wide KO screen that can be used to identify low-affinity interactions mediated by the receptors present on the surface of the cells [67] (unpublished). The field of identification of low-affinity receptor-ligand interactions has been challenging owing to the transient nature of such interactions, which make them difficult to detect in many existing biochemical methods. By using cell lines that express endogenous receptors on the surface of cells and a genome-wide screening approach on the cell lines to identify factors that contribute to binding to recombinant proteins, the approach we have developed allows for the identification of receptor-ligand interactions at the surface of cells. Besides, because of the genome-scale nature of the screen, the method also facilitates the study of the molecular nature and cell biology of surface receptors without the need to make any prior assumptions regarding their biochemical properties.

Recently, a number of studies has also extended the use of pooled screening from cell-lines to whole animal models to interrogate in vivo signalling processes mainly in the context of cancer. In vivo screens are commonly performed using xenograft models in mice or less commonly by directly modifying endogenous mouse cells. Such screens have been used to identify cellular signalling pathways that cause resistance to immunotherapy in the context of melanoma [68], to identify liver tumour suppressors [69], to identify suppressors in glioblastoma [70] and to study essential genes in the context of mutant KRAS colorectal cancers [71]. While the use of in vivo screens at a genome-wide scale is still limited by the challenges relating to maintenance of the library complexity in the animal model, limitations of virus delivery, and technical difficulties in uniformly transducing cells in situ at a low multiplicity of infection (MOI), these screens are powerful tools that can be used to recapitulate the cellular processes in a complex multicellular organism rather than in a relatively simple environment of a cell culture model.

Pooled genome-wide screens, while useful to infer biological function, do not provide detailed information regarding the cell state in terms of its transcriptional status. A readout in a traditional CRISPR/Cas9 pooled screen is simply the abundance of gRNA sequences contained within the pool of cells, which cannot effectively provide the required resolution for comprehensive phenotyping. A recently-described method termed as CRISPR-UMI (unique molecular identifiers) now addresses some of these limitations as it allows analysis of independently-derived single-cell clones, but it still does not report the transcriptional change at a single cell level [72]. An advance in the field of pooled screening has been the development of techniques that now allow the readout of genomic perturbations at a transcriptomic level. Methods like Perturb-Seq [73,74], CROP-Seq [75] and CRISP-Seq [76] and Mosaic-Seq [77] have combined pooled screening with single-cell sequencing methods, which allows for the fine-resolution dissection of biological processes. Such methods have already been used to dissect aspects of cellular signalling including the T-cell receptor signalling pathway in Jurkat cells [75], the transcriptional program in the BMDC response to lipopolysaccharide (LPS) [73] and mammalian unfolded protein response [74] and regulatory circuits of innate immunity [76]. Currently, the field of gene-expression-based perturbation studies has mostly been used for proof-of-principle demonstrations with a fairly small number of genes from specific families (such as transcription factors and enhancers), as the biological and technical noise in single-cell data make the analysis and interpretation of these datasets computationally challenging. However, these methods hold great potential for the study of cellular signalling pathways as they have the ability to introduce combinatorial perturbations and, in addition, measure the outcome of such perturbations at a transcriptomics level providing higher resolution to study the complex signalling processes.

In addition, novel adjustments to the CRISPR screening system such as the mammalian synthetic cellular recorders integrating biological events (mSCRIBE) now enable tracking of the accumulated mutations created by Cas9 [78]. By using a self-targeting guide RNA (stgRNAs), the authors in this work tested the application of this system using NF-kB activation upon stimulation with LPS and were able to show that as the magnitude and the duration of signal input increased, the percent of mutated stgRNA is also increased. This provided an example of how CRISPR-based systems can be used to record the duration and magnitude of biological events, an application of which would provide a novel tool to track the extent of context-dependent signalling pathways.

**Figure 2:**
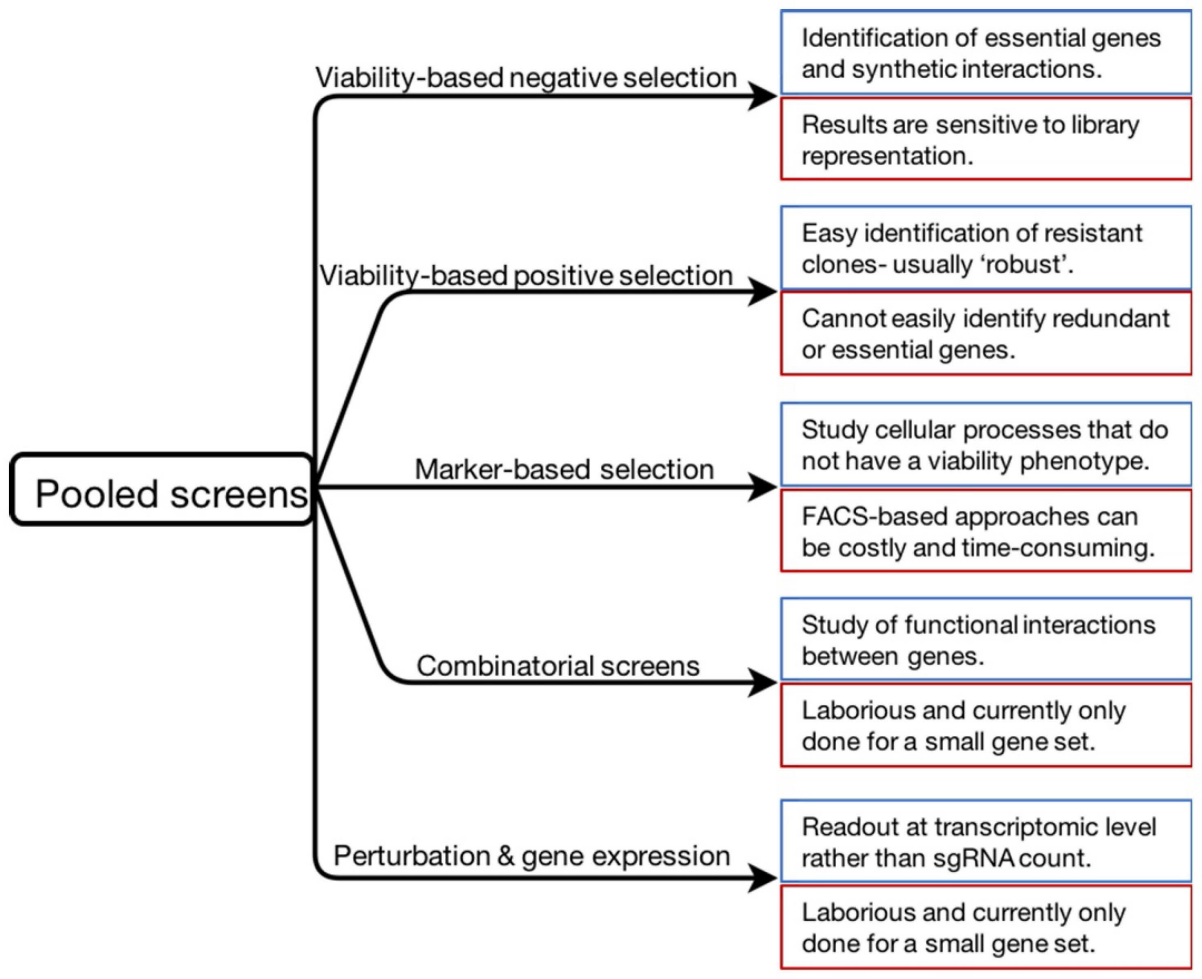
Summary of the screening approaches described in this review. Specific features of each approach are indicated in blue (strengths) and red (limitations) boxes.

The studies reviewed here show the diversity of ways in which CRISPR/Cas9 screens can now be used to understand biological processes (summarized in Figure 2). CRISPR/Cas9-based perturbation studies therefore hold great promise for the systematic dissection of complex processes, including the cellular signalling process.

## Abbreviations

AKT	Protein kinase B
BMDC	Bone marrow-derived dendritic cells
cAMP	Cyclic adenosine monophosphate
CRISPR/Cas9	Clustered regularly-interspaced short palindromic repeats/Cas9
Erk	Extracellular signal-regulated protein or externally-regulated kinases
EGFR	Epidermal growth factor receptor
FACS	Fluorescence-activated cell sorting
FGF	Fibroblast growth factor
GFP	Green fluorescent protein
GPCR	G-protein–coupled receptors
JNK	Jun N-terminal kinase
JAK	Janus Kinase
LPS	Lipopolysaccharide
MAPK	Mitogen-activated protein kinase
NICD	Notch intracellular domain
NFκB	Nuclear factor κB
OST	Oligosaccharyltransferase
PDGF	Platelet-derived growth factor
PI3K	Phosphatidylinositol 3 kinase
RNAi	RNA interference
RTK	Receptor tyrosine kinase
SAM	Synergistic activation mediator
SHH	Sonic Hedgehog
Smads	Sma and mad homology signal proteins
TGFβ	Transforming growth factor beta
TNF	Tumour necrosis factor

## References

[1] Hunter T. (2000). Signaling-2000 and beyond. Cell.

[2] Finkel T., Silvio Gutkind J. (2003). Signal Transduction and Human Disease.

[3] Deribe Y.L., Pawson T., Dikic I. (2010). Post-translational modifications in signal integration. Nat. Struct. Mol. Biol..

[4] Kim H.D., Shay T., O’Shea E.K., Regev A. (2009). Transcriptional regulatory circuits: Predicting numbers from alphabets. Science.

[5] Amit I., Regev A., Hacohen N. (2011). Strategies to discover regulatory circuits of the mammalian immune system. Nat. Rev. Immunol..

[6] Beyer A., Bandyopadhyay S., Ideker T. (2007). Integrating physical and genetic maps: from genomes to interaction networks. Nat. Rev. Genet..

[7] Choudhary C., Mann M. (2010). Decoding signalling networks by mass spectrometry-based proteomics. Nat. Rev. Mol. Cell Biol..

[8] Spurrier B., Ramalingam S., Nishizuka S. (2008). Reverse-phase protein lysate microarrays for cell signalling analysis. Nat. Protoc..

[9] Jackson A.L., Linsley P.S. (2010). Recognizing and avoiding siRNA off-target effects for target identification and therapeutic application. Nat. Rev. Drug Discov..

[10] Boettcher M., McManus M.T. (2015). Choosing the Right Tool for the Job: RNAi, TALEN, or CRISPR. Mol. Cell.

[11] Doudna J.A., Charpentier E. (2014). The new frontier of genome engineering with CRISPR-Cas9. Science.

[12] Berridge M.J. (2014). Module 2: Cell Signalling Pathways. Cell Signal. Biol..

[13] Doench J.G. (2018). Am I ready for CRISPR? A user’s guide to genetic screens. Nat. Rev. Genet..

[14] Joung J., Konermann S., Gootenberg J.S., Abudayyeh O.O., Platt R.J., Brigham M.D., Sanjana N.E., Zhang F. (2017). Genome-scale CRISPR-Cas9 knock-out and transcriptional activation screening. Nat. Protoc..

[15] Li W., Xu H., Xiao T., Cong L., Love M.I., Zhang F., Irizarry R.A., Liu J.S., Brown M., Liu X.S. (2014). MAGeCK enables robust identification of essential genes from genome-scale CRISPR/Cas9 knock-out screens. Genome Biol..

[16] Hart T., Moffat J. (2016). BAGEL: A computational framework for identifying essential genes from pooled library screens. BMC Bioinform..

[17] Winter J., Breinig M., Heigwer F., Brügemann D., Leible S., Pelz O., Zhan T., Boutros M. (2016). caRpools: An R package for exploratory data analysis and documentation of pooled CRISPR/Cas9 screens. Bioinformatics.

[18] Lo A., Qi L. (2017). Genetic and epigenetic control of gene expression by CRISPR-Cas systems. F1000Research.

[19] Tzelepis K., Koike-Yusa H., De Braekeleer E., Li Y., Metzakopian E., Dovey O.M., Mupo A., Grinkevich V., Li M., Mazan M. (2016). A CRISPR Dropout Screen Identifies Genetic Vulnerabilities and Therapeutic Targets in Acute Myeloid Leukemia. Cell Rep..

[20] Hart T., Chandrashekhar M., Aregger M., Steinhart Z., Brown K.R., MacLeod G., Mis M., Zimmermann M., Fradet-Turcotte A., Sun S. (2015). High-Resolution CRISPR Screens Reveal Fitness Genes and Genotype-Specific Cancer Liabilities. Cell.

[21] Wang T., Birsoy K., Hughes N.W., Krupczak K.M., Post Y., Wei J.J., Lander E.S., Sabatini D.M. (2015). Identification and characterization of essential genes in the human genome. Science.

[22] Tsherniak A., Vazquez F., Montgomery P.G., Weir B.A., Kryukov G., Cowley G.S., Gill S., Harrington W.F., Pantel S., Krill-Burger J.M. (2017). Defining a Cancer Dependency Map. Cell.

[23] Chen L., Alexe G., Dharia N.V., Ross L., Iniguez A.B., Conway A.S., Wang E.J., Veschi V., Lam N., Qi J. (2018). CRISPR-Cas9 screen reveals a MYCN-amplified neuroblastoma dependency on EZH2. J. Clin. Investig..

[24] Giancotti F.G. (2014). Deregulation of cell signalling in cancer. FEBS Lett..

[25] Sever R., Brugge J.S. (2015). Signal transduction in cancer. Cold Spring Harb. Perspect. Med..

[26] Steinhart Z., Pavlovic Z., Chandrashekhar M., Hart T., Wang X., Zhang X., Robitaille M., Brown K.R., Jaksani S., Overmeer R. (2016). Genome-wide CRISPR screens reveal a Wnt–FZD5 signalling circuit as a druggable vulnerability of RNF43-mutant pancreatic tumours. Nat. Med..

[27] O’Neil N.J., Bailey M.L., Hieter P. (2017). Synthetic lethality and cancer. Nat. Rev. Genet..

[28] Wang T., Yu H., Hughes N.W., Liu B., Kendirli A., Klein K., Chen W.W., Lander E.S., Sabatini D.M. (2017). Gene Essentiality Profiling Reveals Gene Networks and Synthetic Lethal Interactions with Oncogenic Ras. Cell.

[29] Evers B., Jastrzebski K., Heijmans J.P.M., Grernrum W., Beijersbergen R.L., Bernards R. (2016). CRISPR knock-out screening outperforms shRNA and CRISPRi in identifying essential genes. Nat. Biotechnol..

[30] Munoz D.M., Cassiani P.J., Li L., Billy E., Korn J.M., Jones M.D., Golji J., Ruddy D.A., Yu K., McAllister G. (2016). CRISPR Screens Provide a Comprehensive Assessment of Cancer Vulnerabilities but Generate False-Positive Hits for Highly Amplified Genomic Regions. Cancer Discov..

[31] Aguirre A.J., Meyers R.M., Weir B.A., Vazquez F., Zhang C.Z., Ben-David U., Cook A., Ha G., Harrington W.F., Doshi M.B. (2016). Genomic copy number dictates a gene-independent cell response to CRISPR-Cas9 targeting. Cancer Discov..

[32] Meyers R.M., Bryan J.G., McFarland J.M., Weir B.A., Sizemore A.E., Xu H., Dharia N.V., Montgomery P.G., Cowley G.S., Pantel S. (2017). Computational correction of copy number effect improves specificity of CRISPR–Cas9 essentiality screens in cancer cells. Nat. Genet..

[33] Shalem O., Sanjana N.E., Hartenian E., Shi X., Scott D.A., Mikkelson T., Heckl D., Ebert B.L., Root D.E., Doench J.G. (2014). Genome-scale CRISPR-Cas9 knock-out screening in human cells. Science.

[34] Hou P., Wu C., Wang Y., Qi R., Bhavanasi D., Zuo Z., Dos Santos C., Chen S., Chen Y., Zheng H. (2017). A Genome-Wide CRISPR Screen Identifies Genes Critical for Resistance to FLT3 Inhibitor AC220. Cancer Res..

[35] Koike-Yusa H., Li Y., Tan E.P., Velasco-Herrera M.D.C., Yusa K. (2014). Genome-wide recessive genetic screening in mammalian cells with a lentiviral CRISPR-guide RNA library. Nat. Biotechnol..

[36] Orchard R.C., Wilen C.B., Doench J.G., Baldridge M.T., McCune B.T., Lee Y.C.J., Lee S., Pruett-Miller S.M., Nelson C.A., Fremont D.H. (2016). Discovery of a proteinaceous cellular receptor for a norovirus. Science.

[37] Marceau C.D., Puschnik A.S., Majzoub K., Ooi Y.S., Brewer S.M., Fuchs G., Swaminathan K., Mata M.A., Elias J.E., Sarnow P. (2016). Genetic dissection of Flaviviridae host factors through genome-scale CRISPR screens. Nature.

[38] Savidis G., McDougall W.M., Meraner P., Perreira J.M., Portmann J.M., Trincucci G., John S.P., Aker A.M., Renzette N., Robbins D.R. (2016). Identification of Zika Virus and Dengue Virus Dependency Factors using Functional Genomics. Cell Rep..

[39] Zhang R., Miner J.J., Gorman M.J., Rausch K., Ramage H., White J.P., Zuiani A., Zhang P., Fernandez E., Zhang Q. (2016). A CRISPR screen defines a signal peptide processing pathway required by flaviviruses. Nature.

[40] Haga K., Fujimoto A., Takai-Todaka R., Miki M., Doan Y.H., Murakami K., Yokoyama M., Murata K., Nakanishi A., Katayama K. (2016). Functional receptor molecules CD300lf and CD300ld within the CD300 family enable murine noroviruses to infect cells. Proc. Natl. Acad. Sci. USA.

[41] Park R.J., Wang T., Koundakjian D., Hultquist J.F., Lamothe-Molina P., Monel B., Schumann K., Yu H., Krupzcak K.M., Garcia-Beltran W. (2017). A genome-wide CRISPR screen identifies a restricted set of HIV host dependency factors. Nat. Genet..

[42] Ma H., Dang Y., Wu Y., Jia G., Anaya E., Zhang J., Abraham S., Choi J.G., Shi G., Qi L. (2015). A CRISPR-Based Screen Identifies Genes Essential for West-Nile-Virus-Induced Cell Death. Cell Rep..

[43] Puschnik A.S., Majzoub K., Ooi Y.S., Carette J.E. (2017). A CRISPR toolbox to study virus-host interactions. Nat. Rev. Microbiol..

[44] Krall E.B., Wang B., Munoz D.M., Ilic N., Raghavan S., Niederst M.J., Yu K., Ruddy D.A., Aguirre A.J., Kim J.W. (2017). KEAP1 loss modulates sensitivity to kinase targeted therapy in lung cancer. eLife.

[45] Terai H., Kitajima S., Potter D.S., Matsui Y., Gutierrez Quiceno L., Chen T., Kim T.J., Rusan M., Thai T.C., Piccioni F. (2017). ER stress signalling promotes the survival of cancer ‘persister cells’ tolerant to EGFR tyrosine kinase inhibitors. Cancer Res..

[46] Konermann S., Brigham M.D., Trevino A.E., Joung J., Abudayyeh O.O., Barcena C., Hsu P.D., Habib N., Gootenberg J.S., Nishimasu H. (2015). Genome-scale transcriptional activation by an engineered CRISPR-Cas9 complex. Nature.

[47] Wang B., Krall E.B., Aguirre A.J., Kim M., Widlund H.R., Doshi M.B., Sicinska E., Sulahian R., Goodale A., Cowley G.S. (2017). ATXN1L, CIC, and ETS Transcription Factors Modulate Sensitivity to MAPK Pathway Inhibition. Cell Rep..

[48] Kong X., Kuilman T., Shahrabi A., Boshuizen J., Kemper K., Song J.Y., Niessen H.W.M., Rozeman E.A., Geukes Foppen M.H., Blank C.U. (2017). Cancer drug addiction is relayed by an ERK2-dependent phenotype switch. Nature.

[49] Breslow D.K., Hoogendoorn S., Kopp A.R., Morgens D.W., Vu B.K., Kennedy M.C., Han K., Li A., Hess G.T., Bassik M.C. (2018). A CRISPR-based screen for Hedgehog signalling provides insights into ciliary function and ciliopathies. Nat. Genet..

[50] Patel S.J., Sanjana N.E., Kishton R.J., Eidizadeh A., Vodnala S.K., Cam M., Gartner J.J., Jia L., Steinberg S.M., Yamamoto T.N. (2017). Identification of essential genes for cancer immunotherapy. Nature.

[51] Parnas O., Jovanovic M., Eisenhaure T.M., Herbst R.H., Dixit A., Ye C.J., Przybylski D., Platt R.J., Tirosh I., Sanjana N.E. (2015). A Genome-wide CRISPR Screen in Primary Immune Cells to Dissect Regulatory Networks. Cell.

[52] DeJesus R., Moretti F., McAllister G., Wang Z., Bergman P., Liu S., Frias E., Alford J., Reece-Hoyes J.S., Lindeman A. (2016). Functional CRISPR screening identifies the ufmylation pathway as a regulator of SQSTM1/p62. eLife.

[53] Potting C., Crochemore C., Moretti F., Nigsch F., Schmidt I., Manneville C., Carbone W., Knehr J., DeJesus R., Lindeman A. (2018). Genome-wide CRISPR screen for PARKIN regulators reveals transcriptional repression as a determinant of mitophagy. Proc. Natl. Acad. Sci. USA.

[54] Pusapati G.V., Kong J.H., Patel B.B., Krishnan A., Sagner A., Kinnebrew M., Briscoe J., Aravind L., Rohatgi R. (2018). CRISPR Screens Uncover Genes that Regulate Target Cell Sensitivity to the Morphogen Sonic Hedgehog. Dev. Cell.

[55] Lebensohn A.M., Dubey R., Neitzel L.R., Tacchelly-Benites O., Yang E., Marceau C.D., Davis E.M., Patel B.B., Bahrami-Nejad Z., Travaglini K.J. (2016). Comparative genetic screens in human cells reveal new regulatory mechanisms in WNT signalling. eLife.

[56] Billmann M., Chaudhary V., ElMaghraby M.F., Fischer B., Boutros M. (2018). Widespread Rewiring of Genetic Networks upon Cancer Signaling Pathway Activation. Cell Syst..

[57] Horn T., Sandmann T., Fischer B., Axelsson E., Huber W., Boutros M. (2011). Mapping of signalling networks through synthetic genetic interaction analysis by RNAi. Nat. Methods.

[58] Wong A.S.L., Choi G.C.G., Cui C.H., Pregernig G., Milani P., Adam M., Perli S.D., Kazer S.W., Gaillard A., Hermann M. (2016). Multiplexed barcoded CRISPR-Cas9 screening enabled by CombiGEM. Proc. Natl. Acad. Sci. USA.

[59] Wong A.S.L., Choi G.C.G., Cheng A.A., Purcell O., Lu T.K. (2015). Massively parallel high-order combinatorial genetics in human cells. Nat. Biotechnol..

[60] Han K., Jeng E.E., Hess G.T., Morgens D.W., Li A., Bassik M.C. (2017). Synergistic drug combinations for cancer identified in a CRISPR screen for pairwise genetic interactions. Nat. Biotechnol..

[61] Shen J.P., Zhao D., Sasik R., Luebeck J., Birmingham A., Bojorquez-Gomez A., Licon K., Klepper K., Pekin D., Beckett A.N. (2017). Combinatorial CRISPR-Cas9 screens for de novo mapping of genetic interactions. Nat. Methods.

[62] Du D., Roguev A., Gordon D.E., Chen M., Chen S.H., Shales M., Shen J.P., Ideker T., Mali P., Qi L.S. (2017). Genetic interaction mapping in mammalian cells using CRISPR interference. Nat. Methods.

[63] Dahlman J.E., Abudayyeh O.O., Joung J., Gootenberg J.S., Zhang F., Konermann S. (2015). Orthogonal gene knock-out and activation with a catalytically active Cas9 nuclease. Nat. Biotechnol..

[64] Boettcher M., Tian R., Blau J.A., Markegard E., Wagner R.T., Wu D., Mo X., Biton A., Zaitlen N., Fu H. (2018). Dual gene activation and knock-out screen reveals directional dependencies in genetic networks. Nat. Biotechnol..

[65] Tanenbaum M.E., Gilbert L.A., Qi L.S., Weissman J.S., Vale R.D. (2014). A protein-tagging system for signal amplification in gene expression and fluorescence imaging. Cell.

[66] Unniyampurath U., Pilankatta R., Krishnan M.N. (2016). RNA Interference in the Age of CRISPR: Will CRISPR Interfere with RNAi?. Int. J. Mol. Sci..

[67] Sharma S., Bartholdson S.J., Couch A.C., Yusa K., Wright G.J. Genome-Scale Identification of Cellular Pathways Required for Cell Surface Recognition.

[68] Manguso R.T., Pope H.W., Zimmer M.D., Brown F.D., Yates K.B., Miller B.C., Collins N.B., Bi K., LaFleur M.W., Juneja V.R. (2017). In vivo CRISPR screening identifies Ptpn2 as a cancer immunotherapy target. Nature.

[69] Song C.Q., Li Y., Mou H., Moore J., Park A., Pomyen Y., Hough S., Kennedy Z., Fischer A., Yin H. (2017). Genome-Wide CRISPR Screen Identifies Regulators of Mitogen-Activated Protein Kinase as Suppressors of Liver Tumors in Mice. Gastroenterology.

[70] Chow R.D., Guzman C.D., Wang G., Schmidt F., Youngblood M.W., Ye L., Errami Y., Dong M.B., Martinez M.A., Zhang S. (2017). AAV-mediated direct in vivo CRISPR screen identifies functional suppressors in glioblastoma. Nat. Neurosci..

[71] Yau E.H., Kummetha I.R., Lichinchi G., Tang R., Zhang Y., Rana T.M. (2017). Genome-Wide CRISPR Screen for Essential Cell Growth Mediators in Mutant KRAS Colorectal Cancers. Cancer Res..

[72] Michlits G., Hubmann M., Wu S.H., Vainorius G., Budusan E., Zhuk S., Burkard T.R., Novatchkova M., Aichinger M., Lu Y. (2017). CRISPR-UMI: single-cell lineage tracing of pooled CRISPR-Cas9 screens. Nat. Methods.

[73] Dixit A., Parnas O., Li B., Chen J., Fulco C.P., Jerby-Arnon L., Marjanovic N.D., Dionne D., Burks T., Raychowdhury R. (2016). Perturb-Seq: Dissecting Molecular Circuits with Scalable Single-Cell RNA Profiling of Pooled Genetic Screens. Cell.

[74] Adamson B., Norman T.M., Jost M., Cho M.Y., Nuñez J.K., Chen Y., Villalta J.E., Gilbert L.A., Horlbeck M.A., Hein M.Y. (2016). A Multiplexed Single-Cell CRISPR Screening Platform Enables Systematic Dissection of the Unfolded Protein Response. Cell.

[75] Datlinger P., Rendeiro A.F., Schmidl C., Krausgruber T., Traxler P., Klughammer J., Schuster L.C., Kuchler A., Alpar D., Bock C. (2017). Pooled CRISPR screening with single-cell transcriptome readout. Nat. Methods.

[76] Jaitin D.A., Weiner A., Yofe I., Lara-Astiaso D., Keren-Shaul H., David E., Salame T.M., Tanay A., van Oudenaarden A., Amit I. (2016). Dissecting Immune Circuits by Linking CRISPR-Pooled Screens with Single-Cell RNA-Seq. Cell.

[77] Xie S., Duan J., Li B., Zhou P., Hon G.C. (2017). Multiplexed Engineering and Analysis of Combinatorial Enhancer Activity in Single Cells. Mol. Cell.

[78] Perli S.D., Cui C.H., Lu T.K. (2016). Continuous genetic recording with self-targeting CRISPR-Cas in human cells. Science.

